# Structure and binding properties of Pangolin-CoV spike glycoprotein inform the evolution of SARS-CoV-2

**DOI:** 10.1038/s41467-021-21006-9

**Published:** 2021-02-05

**Authors:** Antoni G. Wrobel, Donald J. Benton, Pengqi Xu, Lesley J. Calder, Annabel Borg, Chloë Roustan, Stephen R. Martin, Peter B. Rosenthal, John J. Skehel, Steven J. Gamblin

**Affiliations:** 1 Structural Biology of Disease Processes Laboratory, Francis Crick Institute, NW1 1AT, London, UK; 2 Precision Medicine Center, The Seventh Affiliated Hospital, Sun Yat-sen University, Shenzhen, Guangdong, China; 3 Structural Biology of Cells and Viruses Laboratory, Francis Crick Institute, NW1 1AT, London, UK; 4 Structural Biology Science Technology Platform, Francis Crick Institute, NW1 1AT, London, UK

**Keywords:** SARS-CoV-2, Cryoelectron microscopy

## Abstract

Coronaviruses of bats and pangolins have been implicated in the origin and evolution of the pandemic SARS-CoV-2. We show that spikes from Guangdong Pangolin-CoVs, closely related to SARS-CoV-2, bind strongly to human and pangolin ACE2 receptors. We also report the cryo-EM structure of a Pangolin-CoV spike protein and show it adopts a fully-closed conformation and that, aside from the Receptor-Binding Domain, it resembles the spike of a bat coronavirus RaTG13 more than that of SARS-CoV-2.

## Abstract (web summary)

It has been suggested that pangolin coronaviruses may be the origin of SARS-CoV-2. Here the authors show that the Pangolin-CoV spike is structurally closely related to the closed form of SARS-CoV-2 spike and exhibits similar binding properties to human and pangolin ACE2; although neither spike binds bat ACE2.

## Introduction

Despite intensive research into the origins of the COVID-19 pandemic, the evolutionary history of its causative agent SARS-CoV-2 remains unclear^1,2^. SARS-CoV-2 belongs to the subgenus of sarbecoviruses, for which horseshoe bats (*Rhinolophus sp*.) are the reservoir species^1,3,4^. However, others have suggested^5^ and we recently demonstrated^6^, that the bat coronavirus RaTG13, the closest known relative of SARS-CoV-2, is unlikely to be able to infect human cells because of the very low affinity of its spike protein (S) for the human receptor. For this reason, it has been speculated that SARS-CoV-2 could have reached the human population via an intermediate host^5^. A number of recent studies reported the existence of sarbecoviruses highly similar to SARS-CoV-2 in diseased Malayan pangolins (*Manis javanica*) and thus pangolins were proposed to have played a role in the emergence of the current pandemic^7–10^. Here, we analyse ACE2-binding properties and the structure of S protein from a Pangolin-CoV closely related to SARS-CoV-2^8,9^.

## Results

### The affinity of Pangolin-CoV S proteins for ACE2 receptors

**Fig. 1:**
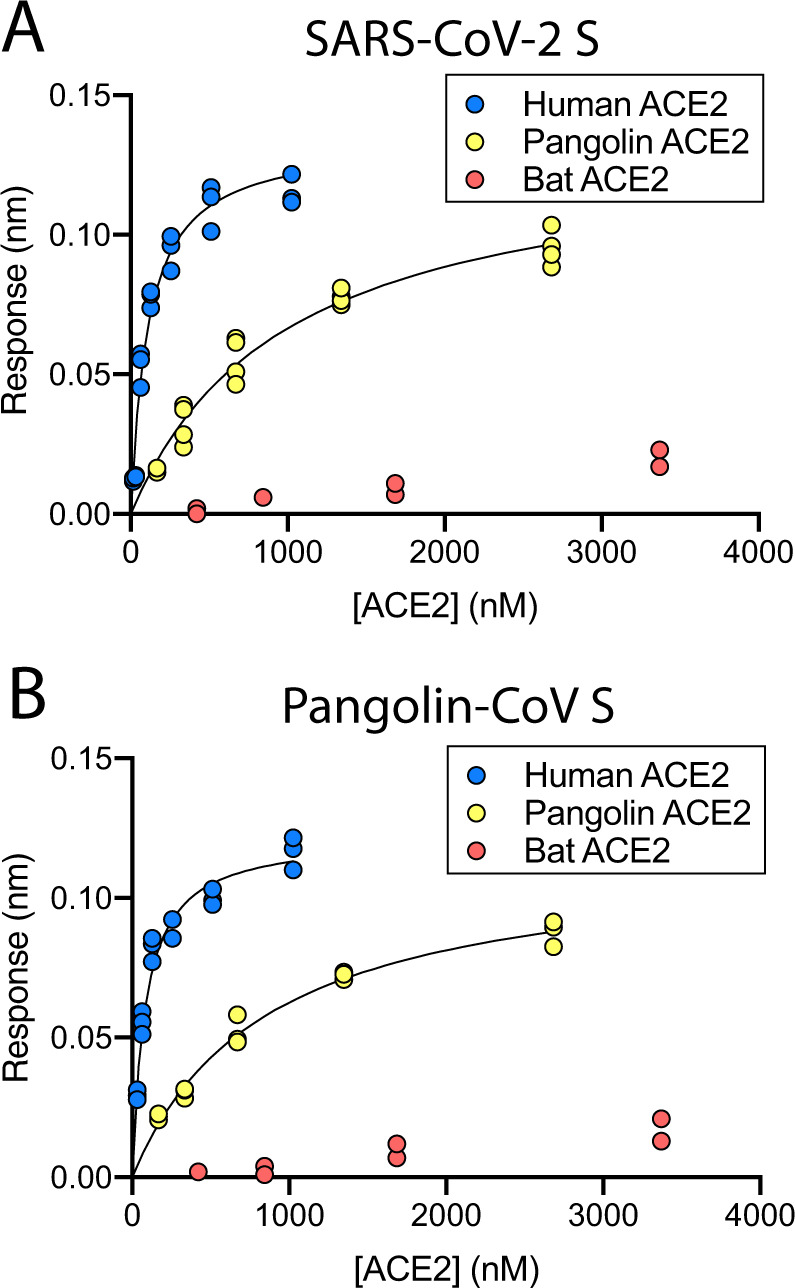
Binding of Pangolin-CoV and SARS-CoV-2 S to ACE2s from different species. Plots of biolayer interferometry amplitudes for human (blue), pangolin (yellow) and bat (red) ACE2s binding to SARS-CoV-2 S (**A**) and Pangolin-CoV S (**B**).

To characterise the pangolin virus spike and compare it with that of SARS-CoV-2, we expressed and purified two different Pangolin-CoV spike ectodomains. These are based on the sequences of viruses isolated from pangolins seized in China’s Guangdong province in 2019^8,9^. We also produced recombinant ectodomains of ACE2 proteins from human, bat (*Rhinolophus ferremequinum*) and pangolin in order to perform comparative biolayer interferometry assays. Both pangolin proteins (referred to as Pangolin-CoV S and Pangolin-CoV S’) showed strong (<100 nM) binding to the human ACE2, approximately ten-fold weaker binding to pangolin ACE2, and very weak binding to bat ACE2 (Fig. 1A and Supplementary Fig. 1). A similar pattern of binding was observed for SARS-CoV-2 S (Fig. 1B); preferred and strong binding to human ACE2, weaker binding to pangolin ACE2 and very weak binding to bat ACE2. The binding of pangolin S to human and pangolin ACE2 is comparable to SARS-CoV-2 S (Table 1 and Supplementary Fig. 1E), in keeping with the very high sequence and structural similarity between their two RBDs (Table 2). None of the three species of ACE2 were bound strongly by the bat virus RaTG13 S. This observation correlates with the substantial sequence differences between the RBD of RaTG13 and the RBDs of spike proteins from the viruses of the other two species (Table 2).

**Table 1:** Binding of Pangolin-CoV and SARS-CoV-2 S to ACE2s from different species.

Spike	ACE2	K_d_ amplitude (nM)	K_d_ kinetics (nM)
SARS-CoV-2	Human	110 ± 14.7	75.5 ± 12.9
	Pangolin	987 ± 112	896 ± 225
Pangolin-CoV	Human	74.0 ± 13.0	42.1 ± 10.0
	Pangolin	850 ± 169	663 ± 139

Equilibrium dissociation constants determined from the analysis of the data in Fig. 1 (K_d_ Amplitude) compared with values determined from analysis of the corresponding kinetic data (K_d_ Kinetics) (see Fig. S1).

**Table 2:** Comparison of RBD sequence identity and RMSD of atom positions.

	SARS-CoV-2	Pangolin-CoV	RaTG13	
SARS-CoV-2	–	0.35	0.87	
Pangolin-CoV	96.5	–	0.62	RMSD of RBD (Å)
RaTG13	89.5	89.0	–	
		RBD Sequence Identity (%)		

Right hand side: RMSD of atom positions in the RBD structures of RaTG13 S (6ZGF)^6^, closed conformation of SARS-CoV-2 S (6ZGE)^6^, and Pangolin-CoV S determined in this study. Left hand side: sequence identity of the RBDs from the same viruses.

### Cryo-EM structure of Pangolin-CoV S

**Fig. 2:**
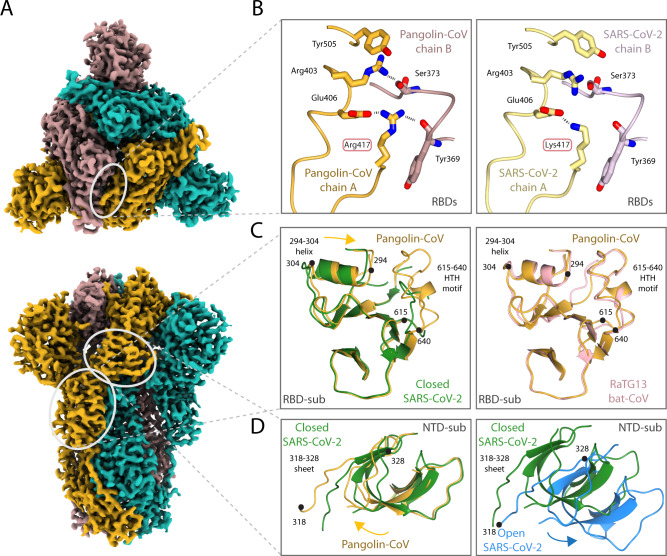
Structure of Pangolin-CoV spike protein. **A** EM density representation from the 2.9 Å map of Pangolin-CoV S viewed from down the three-fold axis (top panel) and in the orthogonal view (lower panel). The subunits are coloured in sea blue, golden and rosy brown. The white ovals identify the areas shown in molecular representation on the right. **B** Comparison of the RBD/RBD interface from the pangolin-CoV (left) and SARS-CoV-2 S (PDB: 6ZGE, right) highlighting the Arg417Lys substitution. **C** Comparison of the RBD-associated subdomains of the pangolin-CoV (golden) and closed form of SARS-CoV-2 (green) in the left hand panel, showing the different positioning of the 294–304 helix and the presence of the 615–640 helix-turn-helix in the pangolin structure and, in the right hand panel, the overlap of the same Pangolin-CoV S structure (golden) with the corresponding region from the RaTG13 (PDB: 6ZGF) (pink). **D** Comparison of (left) the NTD-associated subdomain of Pangolin-CoV (golden) with that of the closed form of SARS-CoV-2 (green) showing the different domain orientations between them; (right) the closed (green) and open (blue) conformations of the NTD-associated subdomain of SARS-CoV-2 showing that the shift in orientation of the NTD-associated subdomain on spike opening is in the opposite direction to the shift seen between the Pangolin-CoV and closed SARS-CoV-2 conformations shown in the left panel.

We have determined the structure of the Pangolin-CoV S protein at 2.9 Å by Cryo-EM (Fig. 2, Table 3, Supplementary Fig. 2). The structure is of similar resolution to our recent structure of SARS-CoV-2 S^6^, enabling a detailed comparison between the two. Overall, the structure of the Pangolin-CoV S (Fig. 2A) is similar to the closed form of the SARS-CoV-2 S and the RaTG13 S; the most striking feature is that all of the resolvable particles on the grid are in the closed conformation (compared with 83% in the uncleaved SARS-CoV-2 S sample and 34% in the furin-cleaved in our previous study^6^). Comparison of the structures of S of Pangolin-CoV and SARS-CoV-2 identifies two amino-acid changes that likely account for this feature.

**Table 3:** Cryo-EM data collection, refinement and validation statistics.

	Pangolin-CoV (EMD-12130) (PDB 7BBH)
*Data collection and processing*	
Voltage (kV)	300
Electron exposure (e–/Å^2^)	51.8
Defocus range (μm)	−1.5 to −3.0
Pixel size (Å)	1.08
Symmetry imposed	C3
Final particle images (no.)	93 k
Map resolution (Å)	2.9
FSC threshold = 0.143	
Map resolution range (Å)	2.8–3.6
*Refinement*	
Initial model used (PDB code)	6ZGE
Model resolution (Å)	3.0
FSC threshold = 0.5	
Map sharpening *B* factor (Å^2^)	−86.5
Model composition	
Non-hydrogen atoms	25,827
Protein residues	3189
Ligands	69
*B* factors (Å^2^)	
Protein	38.0
Ligand	70.9
R.m.s. deviations	
Bond lengths (Å)	0.005
Bond angles (°)	0.707
Validation	
MolProbity score	1.36
Clashscore	3.02
Poor rotamers (%)	0.86
Ramachandran plot	
Favored (%)	96.13
Allowed (%)	3.87
Disallowed (%)	0.00

Firstly, an amino-acid substitution in the otherwise highly conserved sequences in the interface between RBD neighbours in the S trimer, likely contributes to a more stable packing arrangement that favours the closed conformation (Fig. 2B). In detail, there is a salt bridge in the closed form of SARS-CoV-2 formed by Lys417 and Glu406 in the RBD. In the Pangolin-CoV, an arginine is substituted at position 417 and, while it also makes a salt bridge with Glu406, the unique side-chain properties of the arginine residue induce different conformers at Arg403 and Tyr505 that enable additional stacking interactions and the formation of a hydrogen bond to the mainchain of Tyr369 in the neighbouring RBD. These interactions would be expected to contribute additional stabilisation to the RBD/RBD packing, hence favouring the closed form. Furthermore, in Pangolin-CoV S there are also two additional glycans close to the RBD interface (Supplementary Fig. 3).

Secondly, the presence of a leucine residue at position 50 in the NTD-associated intermediate subdomain of Pangolin-CoV, compared with a serine residue in SARS-CoV-2, promotes a conformational arrangement that is further indicative of the closed form of S. Occupancy of a bulky, hydrophobic leucine (instead of the smaller, polar Ser) leads the helix (residues 294–304) to shift 1.5 Å (to the right as viewed in Fig. 2C) compared with SARS-CoV-2, stabilising the formation of a helix-turn-helix structure between the two intermediate domains, which is not present in SARS-CoV-2 S but is present in RaTG13 S (Fig. 2C and Supplementary Fig. 3a). Folding of this motif has the effect of shifting the neighbouring RBD-associated subdomain as a rigid-body (to the left as viewed in Fig. 2D). A similar arrangement, of the helix-turn-helix, and rigid-body position of the domain are seen in the closed conformation of RaTG13 S (Supplementary Fig. 3b). Moreover, analysis of the open conformations of SARS-CoV-2 S shows that the RBD-associated intermediate domain shifts in the opposite direction upon S opening (Fig. 2D).

## Discussion

Taken together, these observations suggest several sequence-based differences, compared with SARS-CoV-2, that likely account for the Pangolin-CoV spike adopting an all-closed conformation. In an earlier work, we described the closed conformation adopted by the bat CoV RaTG13 S protein^6^. In that case, chemical crosslinking was required to stabilise the protein for Cryo-EM analysis, and so the possibility existed that the crosslinking had influenced its structure. The fact that the current closed conformation of Pangolin-CoV S is remarkably similar, outside of the RBD, to the RaTG13 S suggests that the structure of the latter was probably not materially affected by the crosslinking.

The likely role of the closed conformation for shielding the fusion apparatus of S2 has been detailed before^11–13^, and also the need for the open conformation to facilitate receptor binding^14,15^. The similarity in affinity of the pangolin (all closed in cryo-EM) trimeric spike compared with the furin-cleaved SARS-CoV-2 trimeric spike (>60% non-closed in cryo-EM^6^) used in this study (Fig. 1 and Table 1) implies that there is not a large energetic cost to opening of the S1 structure. This notion is further supported by the observation that both the uncleaved (mostly closed^6^) and furin-cleaved SARS-CoV-2 spikes show very similar affinity for ACE2, with Kds determined using the same methodology and calculated from kinetic constants equal to 67.5 ± 9.0^6^ and 75.5 ± 12.9 nM, respectively (Fig. 1, Table 1, and Supplementary Fig. 1). Furthermore, our recent data^16^ have shown that the presence of ACE2 receptors enhances the opening of the RBDs of the SARS-CoV-2 spike and its priming for subsequent membrane fusion. In this way, the more open conformation of the spike of SARS-CoV-2 relative to the pangolin spike, while not leading to tighter binding of ACE2 by the spike, may facilitate an early kinetic event in the binding process that does not affect the eventual equilibrium association values.

The non-RBD component of the S protein of SARS-CoV-2 is very similar to that of the bat virus RaTG13 protein (96% identity within S1). By contrast, their sequence identity is just 76% in the RBD. On the other hand, the sequence (97% identity) and structure (RMSD 0.35 Å, Table 2) of the RBD of SARS-CoV-2, is remarkably similar to that of Pangolin-CoV, particularly at the ACE2-binding site. This close similarity of RBDs between Pangolin-CoV and SARS-CoV-2 correlates with the near identical binding properties of their two S proteins (Fig. 1). This suggests that, even though Pangolin-CoV and SARS-CoV-2 have significant sequence differences beyond their RBDs, especially in the NTD, which for the Pangolin-CoV spikes resembles more that of bat viruses ZXC21 and ZC45 than RaTG13, pangolin viruses might well be capable of infecting humans. In contrast, given the immeasurably low affinity of bat RaTG13 S for human ACE2, it seems unlikely that at least this class of presumed precursor bat viruses would infect humans.

There are conflicting reports on whether the RBD of Pangolin-CoV S, while very similar in sequence to the RBD of the current pandemic virus, is the immediate precursor to the SARS-CoV-2 RBD^17,18^. Our results suggest that the effective zoonotic range for this class of coronaviruses, beyond bats, may include species that, like pangolins, have ACE2 receptors similar to the human ACE2. Consequently, there are likely to be other, as yet unidentified, viruses that harbour RBDs of similar sequence and binding properties to SARS-CoV-2 and Pangolin-CoV. The existence of such RBDs in the relevant zoonotic background might account for the emergence of SARS-CoV-2 possibly via a recombination of bat viruses similar to RaTG13 with viruses perhaps not dissimilar to Pangolin-CoV. It is also important to note that various species of bat, even within the *Rhinolophus* genus, show considerable differences in their ACE2 sequences and that it has not been possible to demonstrate direct binding of spike proteins from the viruses most closely related to SARS-CoV-2 to bat ACE2. Thus, S of bat viruses may bind a different, as yet unidentified, cellular receptor(s).

## Methods

### Protein constructs

The constructs coding for Pangolin-CoV S ectodomains were based on coronavirus sequences reported by two independent groups, both of which isolated virus material from diseased Malayan pangolins (*Manis javanica*) likely smuggled into China’s Guangdong province in 2019. Pangolin-CoV S’ corresponded to residues 1–1200 (the equivalent of 1–1208 for SARS-CoV-2) of the S identified in the Pangolin-CoV genome (GISAID number EPI_ISL_410721) reported by Xiao et al.^8^ and Pangolin-CoV S corresponded to residues 1–1200 (also the 1–1208 equivalent in SARS-CoV-2) of the S (NCBI number QIG55945.1) from the Pangolin coronavirus MP789 isolate reported by Liu et al.^9^. Both constructs were made as “2 P” mutants for greater stability^19^, codon optimised for human expression and cloned by GenScript with the same expression and purification tags as described previously for RaTG13 and SARS-CoV-2 S^6^ viz. the N-terminal secretion sequence derived from μ-phosphatase and a C-terminal tag consisting of a TEV-cleavage site, the foldon trimerisation domain, and a hexahistidine. The RaTG13 and SARS-CoV-2 S (with its furin-cleavage site intact) constructs used here had the same overall architecture and were described previously^6^.

The construct coding for the human ACE2 ectodomain (residues 1–615, NCBI reference NM_021804.2) was codon optimised and made with a C-terminal Twin-strep tag preceded by a DYK-tag and cloned into pcDNA.3.1(+) by GenScript. The ACE2 ectodomains (residues 19–615) from the Malayan pangolin (*Manis javanica*, NCBI reference XP_017505746.1) and an archetypal horseshoe bat species, Greater horseshoe bat (*Rhinolophus ferremequinum*, Uniprot reference B6ZGN7) were also cloned by Genscript into pcDNA.3.1(+) with the same tags as described before for the human ACE2^6^ viz. DYK plus Twin-strep tag at the C-terminus and the secretion leader sequence derived from Ig-kappa at the N-terminus.

### Protein expression and purification

The RaTG13 S, SARS-CoV-2 S, two Pangolin-CoV Spikes (S and S’) and ACE2 ectodomains were made as described before for the SARS-CoV-2 S and human ACE2^6^. Briefly, the proteins were expressed in in Expi293F cells (Gibco) grown in suspension in 37 °C humidified atmosphere with 8% CO_2_. Cells were transfected with 1 mg of DNA per 1 L of cell culture and the protein expressed for 4 (in case of RaTG13 S) or 5 (for ACE2 ectodomains) days. The only difference with the method previously described was that, in case of the SARS-CoV-2 S and Pangolin-CoV S and S’, the cells were transferred to a 32 °C incubator 24 h after the transfection and harvested on the fifth day post transfection for increased yield^20^.

Pangolin-CoV S and S’ were purified using affinity chromatography with TALON beads (Takara), followed by gel filtration into 50 mM MES pH 6.0, 100 mM NaCl buffer on a Superdex 200 Increase 10/300 GL column (GE Life Sciences). SARS-CoV-2 and RaTG13 spikes were made as described previously^6^. SARS-CoV-2 S was not treated with furin in vitro. All three ACE2 ectodomains were purified using Streptactin XT resin (iba) and gel filtered into a buffer containing 20 mM Tris pH 8.0 and 150 mM NaCl as described previously for the human ACE2 ectodomain^6^.

### Biolayer interferometry assays

The biolayer interferometry assays were done as before^6^ using Octet Red 96 (ForteBio) and NiNTA (NTA, ForteBio) sensors in 20 mM Tris pH 8.0, 150 mM NaCl buffer at 25 °C. Spike proteins were immobilised at 20–70 µg/mL concentrations for 45–60 min and ACE2-binding measured using a 120–600 s association and 300–900 s dissociation stages.

Equilibrium dissociation constants (*K*_*d*_) were determined from reaction amplitudes by analysis of the variation of maximum response with ACE2 concentration. *K*_*d*_ values were also determined using analysis of the kinetics of the reactions. Association phases were analysed as a single exponential function, and plots of the observed rate (*k*_obs_) versus ACE2 concentration gave the association and dissociation rate constants (*k*_on_ and *k*_off_) as the slope and intercept, respectively. The *k*_*off*_ values determined in this way were confirmed by analysis of the dissociation phase and *K*_d_ values were determined as *k*_off_/*k*_on_.

### Cryo-EM sample preparation and data collection

Pangolin-CoV S at ~0.15 mg/mL concentration was applied on an R2/2 Quantifoil grid of 200 mesh covered with a thin layer of continuous carbon. The grid was glow discharged for 30 s at 45 mA prior to freezing; 4 uL of the sample was then applied to the grid before it was blotted for between 4 and 4.5 s and plunge frozen into liquid ethane using a Vitrobot MkIII. Data were collected using EPU software on a Titan Krios operating at 300 kV (Thermo Scientific), using a Gatan K2 detector mounted on a Gatan GIF Quantum energy filter operating in zero-loss mode with a slit width of 20 eV. Exposures were of 8 s with an accumulated dose of 51.8 e/Å^2^, which was fractionated into 32 frames. The calibrated pixel size was 1.08 Å and data were collected using a range of defoci between 1.5 and 3 µm.

### Cryo-EM data processing

The frames of collected movies were aligned using MotionCor2^21^, implemented in RELION^22^, with Contrast Transfer Function fitted using CTFfind4^23^. Particles were picked using RELION autopicking, and subjected to 2 rounds of RELION 2D classification, retaining classes with clear secondary structure features. An ab initio 3D model was generated using cryoSPARC^24^ and used as a reference for RELION 3D classification. The particles contained in classes with clear secondary structure were subjected to Bayesian polishing^25^ and refined using cryoSPARC homogeneous refinement, imposing C3 symmetry, with CTF refinement. This generated a map with a global resolution of 2.9 Å. The map had local resolution estimated using blocres^26^ implemented in cryoSPARC, followed by local resolution filtering and global sharpening^27^ in cryoSPARC.

### Model building

The sequence of the Pangolin-CoV S was numbered as for SARS-CoV-2 S (NCBI YP_009724390.1) for the sake of simplicity of comparison. The model was built using our previous structure of SARS-CoV-2 spike (PDB 6ZGE)^6^ as a starting model, with adjustment of the sequence and manual fitting of the model carried out using Coot^28^. Real-space refinement and model validation was carried out using PHENIX^29^.

### Reporting summary

Further information on research design is available in the Nature Research Reporting Summary linked to this article.

## Supplementary information

Supplementary Information

Reporting Summary

## Data Availability

The map and model have been deposited in the Electron Microscopy Data Bank, http://www.ebi.ac.uk/pdbe/emdb/ with accession EMD-12130. The model has been deposited in the Protein Data Bank with acesion code 7BBH [10.2210/pdb7BBH/pdb].
