# Supplementary material for: Structure and binding properties of Pangolin-CoV spike glycoprotein inform the evolution of SARS-CoV-2
Source: Nat Commun. 2021 Feb 5;12:837. doi: 10.1038/s41467-021-21006-9 (PMC7864994; doi:10.1038/s41467-021-21006-9)
Supplement: Supplementary file 1 — Supplementary Information [file 41467_2021_21006_MOESM1_ESM.pdf]

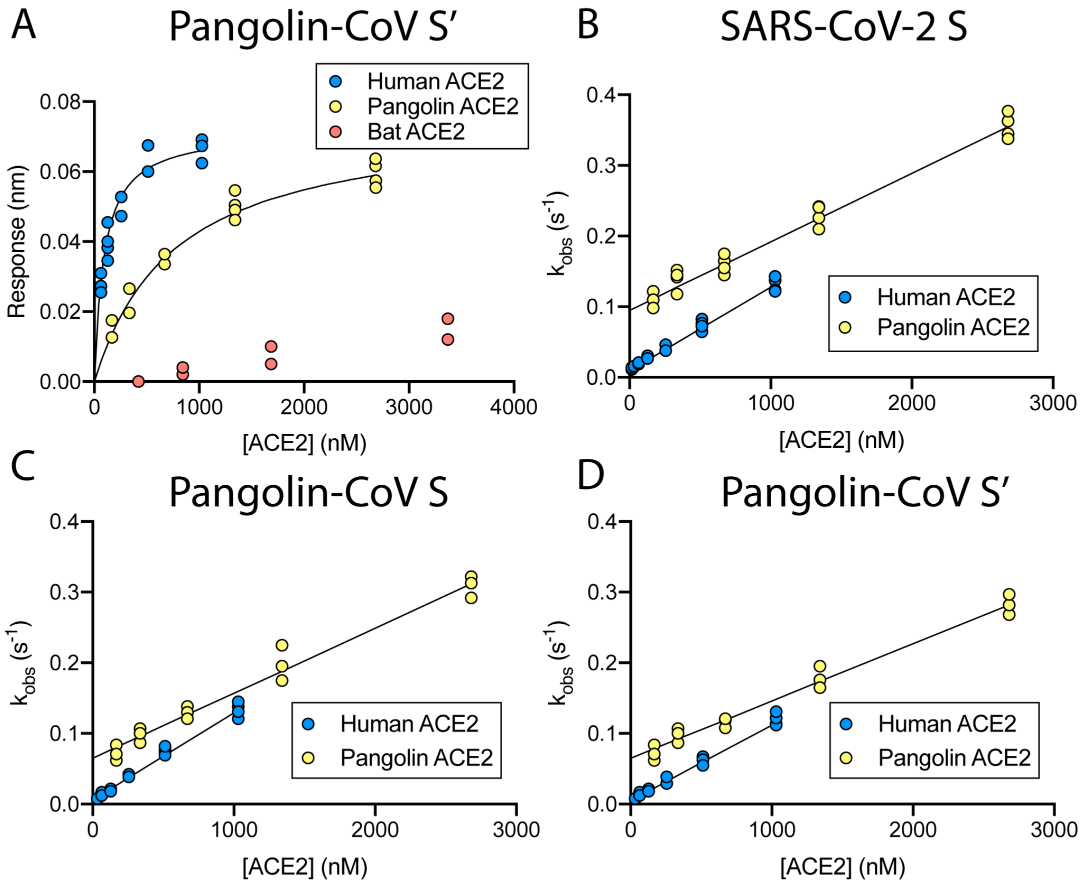

E

Spike	ACE2	k_{on} ($M^{-1}s^{-1}$)	k_{off} (s^{-1})	**K_d Kinetic** (nM)	**K_d Amplitude** (nM)
SARS-CoV-2 S	Human	$1.19x10^5 \pm 3.70x10^3$	$8.99x10^{-3} \pm 1.49x10^{-3}$	75.5 ± 12.9	110.0 ± 14.7
	Pangolin	$9.70x10^4 \pm 6.80x10^3$	$8.70x10^{-2} \pm 2.10x10^{-2}$	896 ± 225	987 ± 112
Pangolin-CoV S	Human	$1.24x10^5 \pm 7.00x10^3$	$5.22x10^{-3} \pm 1.20x10^{-3}$	42.1 ± 10.0	74.0 ± 13.0
	Pangolin	$9.20x10^4 \pm 1.00x10^4$	$6.10x10^{-2} \pm 1.10x10^{-2}$	663.0 ± 140	850 ± 169
Pangolin-CoV S′	Human	$1.08x10^5 \pm 1.50x10^4$	$5.90x10^{-3} \pm 1.50x10^{-3}$	54.6 ± 15.8	82.0 ± 18.0
	Pangolin	$8.10x10^4 \pm 1.10x10^4$	$5.20x10^{-2} \pm 1.70x10^{-2}$	642.0 ± 227	970.0 ± 160

F

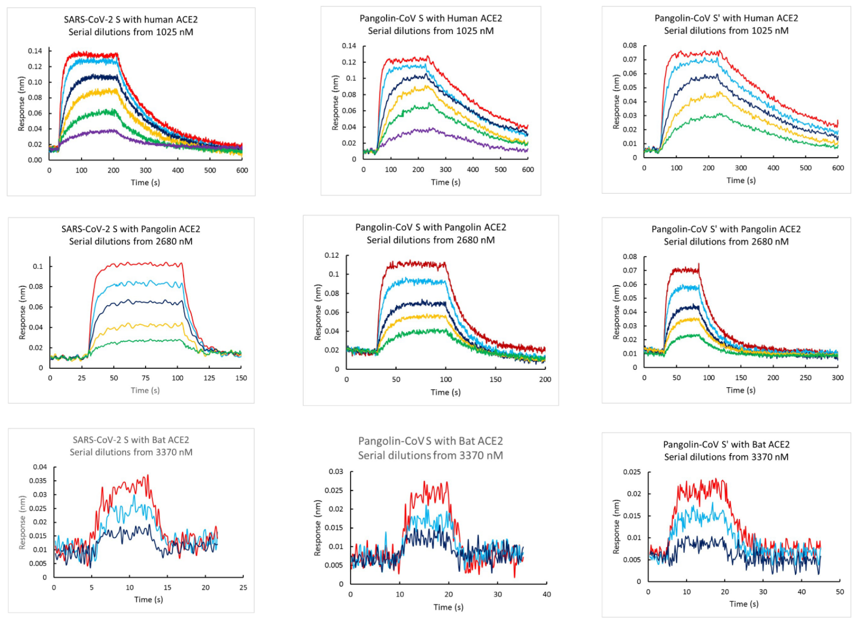

Supplementary Figure 1: Biolayer interferometry on spikes binding to ACE2s from different species. (**A**) Plots of biolayer interferometry amplitudes for human (blue), pangolin (yellow) and bat (red) ACE2s binding to pangolin-CoV S'. (**B, C, D**) Plots of observed rate constants for human (blue) and pangolin (yellow) ACE2s binding to SARS-CoV-2 S (**B**), Pangolin-CoV S (**C**) and Pangolin-CoV S' (**D**). **(E)** Association rate constants, dissociation rate constants and equilibrium dissociation constants. **(F)** Representative traces from biolayer interferometry assays showing binding of ACE2 from human, bat, and pangolin to SARS-CoV-2 S and Pangolin-CoV S' and S. The curves shown are the average of two or more individual curves taken from different experimental sensorgrams. Where necessary the curves were normalised for small variations in spike loading response and corrected for baseline drift as monitored through the behaviour of a reference sensor that was not exposed to ACE2.

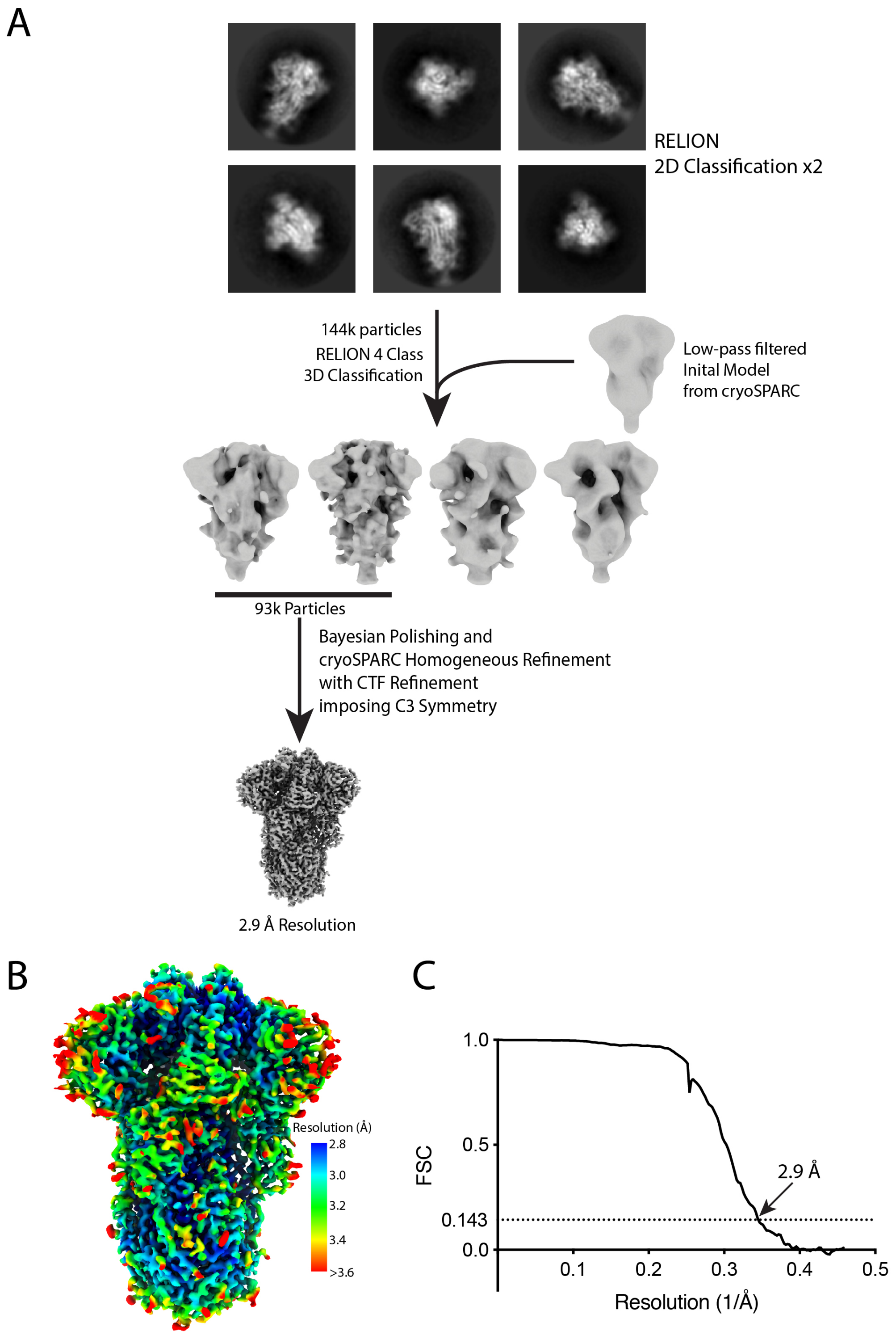

Supplementary Figure 2: EM Data processing. (**A**) Schematic to show steps in cryoEM data processing. (**B**) Final EM density coloured by local resolution. (**C**) Fourier Shell Correlation (FSC) of final EM map.

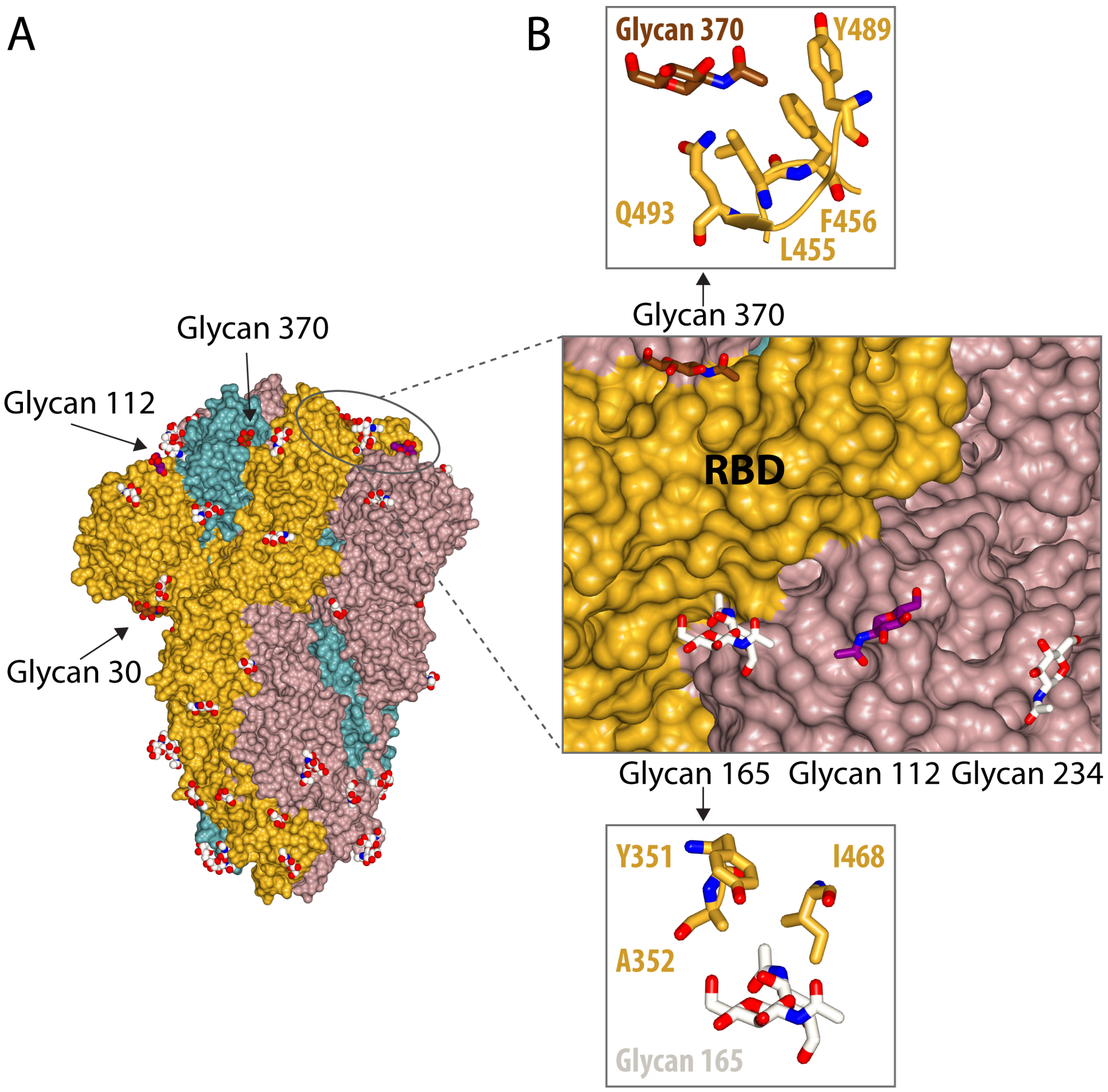

Supplementary Figure 3: N-linked glycosylation of Pangolin-CoV S protein. (A) All glycans present in the Cryo-EM structure of the Pangolin-CoV S shown in balls representation, with those present in RaTG13 S and Pangolin-CoV S, but not SARS-CoV-2, shown in rosy brown and that on Asn112, present uniquely in Pangolin-CoV, in purple. (B) In the middle panel, the glycans on residues 165 and 234, suggested to be important for either inhibiting or enhancing the RBD erection (Henderson et al., 2020), are shown in pale cream, together with the two other glycans 370 and 112, not present in SARS-CoV-2 spike, in proximity of the RBD. The two glycans at residues 370 and 165 of one chain (in pink), which come into direct interaction with the RBD of the neighbouring chain (in yellow) are shown in separate panels, together with the RBD residues they contact.

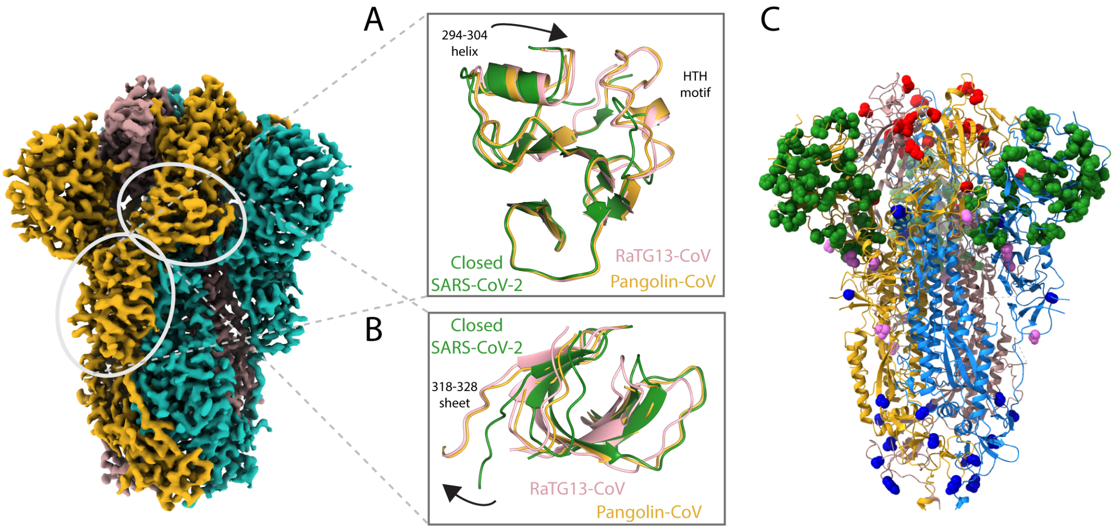

Supplementary Figure 4: Comparison of Pangolin-CoV S with those from RaTG13 and SARS-CoV-2. (**A**, **B**) Panels corresponding to Fig. 2A, 2C (**A**) and 2D (**B**) showing local structure of subdomains of RaTG13 Bat-CoV S (PDB ID 6ZGF, pink) in addition to those of SARS-CoV-2 (PDB ID 6ZGE, green) and Pangolin-CoV (yellow). (**C**) Molecular model of the Pangolin-CoV S protein coloured with monomers in sea blue, golden, and rosy brown in ribbon representation and substitutions between the Pangolin-CoV S and SARS-CoV-2 S shown as spheres in: green (for NTD), light plum (for NTD- and RBD-associated subdomains), red (for RBDs), and navy (S2).

BIBLIOGRAPHY

Henderson, R. *et al.* Glycans on the SARS-CoV-2 Spike Control the Receptor Binding Domain Conformation. *bioRxiv Prepr. Serv. Biol.* 2020.06.26.173765 (2020) doi:10.1101/2020.06.26.173765
